# Whole Animal Experiments Should Be More Like Human Randomized Controlled Trials

**DOI:** 10.1371/journal.pbio.1001481

**Published:** 2013-02-12

**Authors:** Beverly S. Muhlhausler, Frank H. Bloomfield, Matthew W. Gillman

**Affiliations:** 1 FOODplus Research Centre, School of Agriculture Food and Wine, The University of Adelaide, Australia; 2 Liggins Institute, University of Auckland, Auckland, New Zealand; 3 Department of Paediatrics: Child and Youth Health, University of Auckland, Auckland, New Zealand; 4 Gravida, National Centre for Growth and Development, New Zealand; 5 Obesity Prevention Program, Department of Population Medicine, Harvard Medical School and Harvard Pilgrim Health Care Institute, Boston, Massachusetts, United States of America; 6 Department of Nutrition, Harvard School of Public Health, Boston, Massachusetts, United States of America

## Abstract

The quality of reporting of animal studies lags behind that of human randomized controlled trials but a series of additions to the ARRIVE guidelines will help ensure that the standards are comparable.

## Introduction

The reporting of human randomized controlled trials (RCTs) was improved significantly by the introduction of the CONSORT (Consolidated Standards of Reporting Trials) statement in 1996 [1]. CONSORT also led to improvements in the overall quality of human RCTs, benefitting trial design, accounting of subjects, and rigour of data analysis [2],[3]. Whilst human RCTs and whole animal studies may have different objectives (e.g., defining mechanisms versus demonstrating clinical efficacy), the fundamental requirements for generating reliable and unbiased data are very similar, and thus standards of reporting should also be similar. The introduction of the ARRIVE (Animal Research: Reporting In Vivo Experiments) guidelines for conduct and scientific reporting of animal studies in 2010 [4] represented a major step forward in attempting to improve the quality of performing and reporting animal-based research in the same way that the CONSORT statement did for RCTs [1].

Here, we argue that whilst the ARRIVE guidelines are a major step forward, the standards of reporting animal experiments still lag behind those of RCTs. As a result, the validity of results from animal studies and their interpretation are frequently in question. We put forward a series of suggestions for modifying the ARRIVE guidelines to ensure that animal studies catch up. Widespread adoption of these guidelines should improve the overall quality of animal studies, thus improving their relevance to humans.

## Introduction to the CONSORT and ARRIVE Guidelines

Well-designed and conducted human RCTs are widely regarded as providing the top level of scientific evidence for health care interventions (National Health and Medical Research Council of Australia, 2009). The CONSORT statement provides guidelines for reporting the design, conduct, analysis, and interpretation of RCTs and has been adopted by over 400 journals and several key editorial bodies. Its implementation has led to marked improvements in the quality and transparency of reporting of RCTs [2],[3].

In contrast, the reporting of animal studies received comparatively little attention until the publication of the ARRIVE guidelines in 2010 [4]. These guidelines were spurred by a survey of 271 studies reporting original research on rats, mice, and non-human primates carried out in the United Kingdom and the United States of America [5]. The results painted a poor picture of the quality of reporting in animal research. Only 59% of the 271 articles stated the hypothesis or objective of the study, the number of animals used, and characteristics of the animals. Few of the papers surveyed reported using random allocation to treatment group (13%) or blinding of outcome assessment (14%), and statistical methods were not described adequately in 30% of the publications [5]. In a similar review of animal studies published in *Cancer Research*, only 28% reported random allocation of animals to treatment groups, only 2% reported blinding of observers to this allocation, and none reported methods to determine sample size [6]. Similar concerns about underreporting crucial aspects of study design and conduct have been raised by a recent (June 2012) U.S. National Institute of Neurological Disorders and Stroke workshop to “improve the reporting of preclinical studies in grant applications and publications” [7]. The authors of the meeting report emphasized the probable impact that the gap in standards of reporting between animal studies and human clinical trials has had on impairing effective translation from bench to clinic. For example, the false positive rate resulting from poorly performed or reported preclinical experiments may explain why, of the >1,000 treatments investigated for neuroprotection in stroke, none have proved effective clinically [8].

Since 2010, the ARRIVE guidelines have been reprinted by 11 high-impact international journals, and close to 100 scientific journals now include the ARRIVE guidelines in their instructions to authors [9]. The ARRIVE guidelines follow the same general principles as the CONSORT statement and reflect the growing recognition of the need for greater uniformity and accountability in the conduct and reporting of animal-based research, yet they fall short in key areas.

The core elements of both sets of guidelines are presented in Table 1, and in the following paragraphs, we highlight the key reporting elements for well-done RCTs that are not yet included in the ARRIVE guidelines. Specifically, we argue that there is a need for more explicit instructions, particularly in relation to reporting of randomization, blinding, and sample size justification, to ensure that these guidelines are properly implemented and achieve the ultimate aim of improving the design, conduct, and analysis of animal studies, and therefore their usefulness.

**Table 1:** Comparison of the CONSORT and ARRIVE guidelines.

Element	CONSORT	ARRIVE
**Introduction**		
Background and objectives	Scientific background and explanation of rationale (2a)	Include sufficient scientific background (including relevant references to previous work) to understand the motivation and context for the study, and explain the experimental approach and rationale (3a)
		Explain how and why the animal species and model being used can address the scientific objectives and, where appropriate, the study's relevance to human biology (3b)
	Specific objectives or hypotheses (2b)	Clearly describe the primary and any secondary objectives of the study, or specific hypotheses being tested (4)
**Methods**		
Description of study design	Description of trial design (such as parallel, factorial) including allocation ratio (3a)	Indicate number of experimental and control groups (6a); provide precise details of all procedures carried out (7); provide rationale for methods (7d)
	Describe changes to methods after trial commencement (3b)	Describe any modifications to reduce adverse events (16)
Participants	Description of eligibility criteria (4a)	Provide further relevant information such as the source of animals, international strain nomenclature, genetic modification status (e.g., knock-out), genotype, etc. (8b)
	Settings and location of data collection (4b)	State when and where data were collected (7b and c); provide details of housing, husbandry conditions (9a and b)
Interventions	The interventions for each group with sufficient details to allow replication, including how and when they were actually administered (5)	For each experiment and each experimental group, including controls, provide precise details of all procedures carried out (7)
Outcomes	Completely defined prespecified primary and secondary outcome measures, including how and when they were assessed (6a)	Clearly define the primary and secondary experimental outcomes assessed (e.g., cell death, molecular markers, behavioural changes) (12)
	Any changes to trial outcomes after the trial commenced, with reasons (6b)	Not specified
Sample size	How sample size was determined (7a)	Explain how number of animals was arrived at (10b)
	When applicable, explanation of any interim analyses and stopping guidelines (7b)	Not specified
**Randomization**		
Sequence generation	Method used to generate the random allocation sequence (8a)	Not specified
	Type of randomization, including any restriction (blocking or block size) (8b)	Give full details of how animals were allocated to experimental groups, including randomization or matching if done (11a); describe the order in which the animals in the different experimental groups were treated and assessed (11b)
Allocation concealment mechanism	Mechanism used to implement random allocation sequence (details of steps taken to conceal allocation) (9)	Any steps taken to minimise the effects of subjective bias when allocating animals to treatment (e.g., randomization procedure) and when assessing results (e.g., if done, describe who was blinded and when) (6b)
Implementation	Who generated random number sequence, who enrolled participants, who assigned participants to treatments (10)	Not specified
Blinding	Who was blinded after assignment to interventions (11a)	Any steps taken to minimise the effects of subjective bias when assessing results (e.g., if done, describe who was blinded and when) (6b)
**Analysis**		
Statistical methods	Statistical methods used to compare groups for primary and secondary outcomes (12a)	Provide details of statistical methods used for each analysis (13a); specify the unit of analysis for each dataset (13b); describe any methods used to assess whether the data met the assumptions of the statistical approach (13c)
	Methods for additional analyses, such as subgroup analyses and adjusted analyses (12b)	Not specified
Attrition	For each group, the numbers of participants who were randomly assigned, received intended treatment, and were analysed for the primary outcome–flow diagram recommended (13a)	Specify numbers of animals used for each experiment and number in each experimental group (10a); indicate the number of independent replications of each experiment, if relevant (10c)
	For each group, losses and exclusions after randomization, together with reasons (13b)	If not all animals were included, explain why (15b)
Baseline data	Table showing baseline demographic and clinical characteristics of each group (15)	Provide details of the animals used, including species, strain, sex, developmental stage, and weight (8a); report-relevant characteristics and health status of animals prior to treatment or testing (14)
Numbers analysed	For each group, number of participants (denominator) included in each analysis and whether the analysis was by original assigned groups (16)	Report number of animals in each group included in each analysis (15a)
Outcomes and estimation	For each primary and secondary outcome, results for each group, and the estimated effect size and its precision (such as 95% confidence interval) (17a)	Report the results for each analysis carried out, with a measure of precision (e.g., standard error or confidence interval) (16)
	For binary outcomes, presentation of both absolute and relative effect sizes is recommended (17b)	Not specified
Ancillary analyses	Results of any other analyses performed, including subgroup analyses and adjusted analyses, distinguishing prespecified from exploratory (18)	Not specified
Harms	Harms: all important harms or unintended effects in each group (19)	Give details of adverse events in each group (17a)

## Study Setting; Exclusion/Inclusion Criteria

The CONSORT criteria require complete descriptions of the study setting and the eligibility criteria used to select the trial participants [1]. These criteria are critical to assess generalizability of the results. Studies in which the source population is restricted or the eligibility criteria are tight are less likely to be generalizable to a wide swath of patients and populations [10]. In addition, volunteers for most RCTs tend to be healthier than those who do not choose to participate, and thus results may not be generalizable to patients who are less well.

These issues are just as relevant in animal studies. Most animal experiments are conducted on a single breed and strain, which authors almost always report (99% of the studies surveyed by Kilkenny) [5]. However, other inclusion and exclusion criteria, such as age, sex, weight/body condition scores, and health status, are often vague or unreported [5]. The ARRIVE guidelines currently have minimal requirements in this area (Table 1; “Participants”). In addition, most animal researchers have clear ideas about the “quality” of animals that they choose to include, but they typically do not report these quality criteria, how they apply them, or how many animals they excluded based on these criteria. In the same way that RCTs often have a “volunteer bias,” results of animal experiments may not apply even to the same age, sex, and strain if the investigator chooses only the healthiest animals on which to intervene.

## Run-In Period

In RCTs that address efficacy, investigators will often exclude otherwise eligible participants who fail a run-in period (i.e., a period to test their short-term ability to adhere to the treatment regimen irrespective of group assignment). The purpose is to maximize the number of participants who take a “full dose” of intervention as well as return for follow-up assessments throughout the intervention period. Investigators often employ similar “run-in” or acclimatization periods in animal studies, most commonly to assess the response of individual animals to a particular nutritional regimen or surgical procedure. However, even if authors refer to such an acclimatization period, they rarely if ever detail the number and characteristics of animals who fail the run-in. Run-in, or acclimatization, periods may increase the internal validity of results, but they also typically reduce generalizability.

## Randomization

RCTs are distinguished from observational studies by the process of random allocation to treatment group, which, if done properly on an adequately large sample, minimizes confounding. Confounding refers to the nuisance effect of a third variable obscuring the true association between exposure and outcome, and it is the one inherent potential limitation of all observational studies. Randomization equalizes both measured and unmeasured confounders across treatment groups, isolating the experimental treatment as the only difference between them.

### Random Allocation

To be successful, random allocation must be truly random, and most RCTs now use a computer-generated random sequence of numbers to assign treatment status. In contrast, there is very little emphasis randomization technique, or its reporting, in animal research. None of the 271 animal-based papers reviewed by Kilkenny provided adequate details of the randomization procedure [5]. The ARRIVE guidelines are not explicit in requesting reporting of full details of allocation method, including methods of randomization (Table 1; “Randomization”). Adding this reporting requirement is likely to encourage more robust allocation methods in animal studies, minimizing risk of confounding.

### Reporting of Baseline Characteristics

Success of randomization can be verified by reporting a range of baseline characteristics that could potentially confound the observed results, according to treatment assignment [10]. Whilst the majority of the studies surveyed by Kilkenny (2009) stated the sex (74%) and either the age or weight (76%) of the animals overall, these characteristics were not broken down by treatment group [5]. Rarely, if ever, do animal experimenters report anything but a few specific baseline characteristics by treatment group. The ARRIVE guidelines call for reporting baseline data but do not specify reporting according to treatment assignment, which is necessary to assess the success of randomization.

## Blinding (Masking)

As reflected in CONSORT, the participants and all personnel who perform assessments in an RCT should be unaware of treatment assignment [1],[10]. Blinding—whether on the part of participant or staff—helps to ensure that measured treatment response is not affected by conscious or unconscious bias, or any other factor unrelated to the biological action of the treatment. It is preferable for RCT participants to be blinded to the hypothesis of the study, for the same reasons. In addition, in most RCTs, investigators do not unmask the treatment assignment until the experiment is complete, so as not to bias data collection or analysis during the study period.

Kilkenny's 2009 survey reported that 86% of animal studies did not include any report of blinding [5]. While blinding of participants is certainly not as pertinent in animal experiments as in RCTs, blinding of data assessors to treatment assignment is. Even so-called objective measures, such as weight and blood pressure, are subject to systematically inaccurate observation [11]. Many animal studies employ a small team, often involving postgraduate students or junior postdoctoral staff who are responsible for treatment administration, assessment of outcomes, and analysis of data. Having intervention staff also perform outcome assessments and analyse data is contrary to best practice and is likely to increase bias. Thus, we suggest that ARRIVE guidelines (Table 1) call for authors to report how personnel who performed randomization, collected and cleaned data, and analysed results were blinded to treatment allocation, thereby encouraging researchers to follow this critical practice.

## Attrition

In almost all RCTs, the number with outcome assessments is lower than the number randomized [10]. This loss to follow-up, or attrition, can have a number of causes, including inability of the research team to contact participants or to carry out particular assessments. Attrition rates of more than 20% are generally cause for concern, as large loss to follow-up can introduce selection bias. Accurately documenting the progress of all participants through the study, from randomization through data collection, is a key element of the CONSORT statement [1],[12]. A key principle of RCTs is “once in, always in.” Intent-to-treat analysis follows this principle and is the cornerstone of data analysis of RCTs. The inverse is also true: an investigator cannot replace a participant who died or is otherwise lost to follow-up with a new participant.

In animal studies attrition is also common. Kilkenny reported that only 198 of the 271 papers reported animal numbers in both the Methods and Results sections. Importantly, of these 198 papers, 69 (35%) either failed to report clearly the number of animals enrolled and followed up or reported different animal numbers in the results from those in the methods [5]. In the majority of discrepant cases, numbers in the Results section exceeded those in the Methods section, without any explanation from the authors. The ARRIVE guidelines currently advise reporting numbers of animals and reasons for exclusion at baseline. We suggest the guidelines be strengthened to include the number of animals in each group at outcome assessment as well, the reasons for any attrition or missing data elements, and as in RCTs, a comparison of baseline characteristics in animals followed to the end of the study versus those who dropped out.

Authors should follow the same guidelines for each separate analysis, including method (random allocation?) of selection of animals for subgroup comparisons. A flow-chart that details progress of animals through the experiment(s) would improve the transparency of reporting and aid interpretation. Analogous to the RCT, animal experiments should hew to the intent-to-treat principle in data analyses, and any revised ARRIVE guidelines should include a requirement for authors to report how they achieved this goal.

## Adverse Events

The reporting of adverse events is a critical part of RCTs to ensure safety of the intervention being tested [1]. Likewise in animal experiments, animal welfare is a key concern, and adverse events may tip the balance of benefit and risk for the intervention being tested. A serious adverse event may influence further studies on the same intervention; a serendipitous finding may open a whole new avenue of research. ARRIVE guidelines advise reporting of details of adverse events, representing a step forward in recognising the importance of this information [4]. As in human RCTs, animal investigators should design protocols and instruments to detect adverse events with the same rigor as beneficial events. However, any unexpected outcomes associated with a treatment (whether adverse or not) should also be reported.

## Sample Size Issues

In RCTs, calculating the sample size *a priori* ensures sufficient statistical power. The calculation is based on an arbitrary alpha level (usually 0.05), a clinically important or detectable difference in outcome between the treatment arms, and the expected variance if the outcome is a continuous variable. Typical targets for power are 80% (or 90%)—that is, a sample size large enough such that there is no more than a 20% (or 10%) probability that the study will fail to detect an effect when one truly exists [10]. Sample size justification before the RCT begins is an important element of CONSORT (Table 1). It is also important to recognize that once data are collected, the confidence interval provides the needed information on precision of estimates. Power calculations are for study planning, confidence intervals for study reporting [13].

In contrast to RCTs, authors of animal studies rarely report how they arrived at the number of animals in the study and typically do not report confidence intervals. None of the papers included in Kilkenny's review provided any details of sample size calculations [5]. Fortunately, the ARRIVE guidelines require researchers to “explain how the number of animals was arrived at” [4]. However, we believe that these guidelines should go further and stipulate that investigators report how they determined the sample size *a priori*. The alternative, adding animals until “statistical significance” appears, is usually a highly biased approach as it violates principles of random allocation and blinding. We also believe that animal researchers should report confidence intervals in addition to (or instead of) *p* values. The most important results in any study are the effect estimate and its precision. Whether the *p* value is less than an arbitrary value such as 0.05 is unimportant [14].

## Missing Data

Most clinical studies contain some missing data on participants because investigators were unable to collect a piece of information or they excluded outlying (“erroneous”) data points. Identifying erroneous values involves setting rigorous criteria, ideally *a priori*. Criteria may include a range of acceptability for a particular variable, based on prior knowledge of the normal range within the population. If researchers set the range before data collection, then they have the opportunity to repeat the measurement if it falls outside the range, thus minimizing outliers in the final data set.

After data collection, the process involves reviewing and excluding individual data points based on biological plausibility and/or agreement with values from other participants [10]. Investigators should apply predefined rules during the data-cleaning phase, highlighting outlying values and enabling decisions (blinded to treatment group) on whether specific data points are erroneous. It may be possible to verify some data queries by reviewing the source data or, in the case of RCTs, by contacting the participant.

In animal studies these processes should be the same, except that no analogy to contacting participants exists. Animal experimentalists rarely set *a priori* criteria for reasonable ranges for outcome measures, even though it is entirely possible. Moreover, data cleaning is most commonly performed by individuals who are not blinded to the treatment group. Reviewing potentially erroneous data in a blinded manner is crucial. ARRIVE should require researchers to report the procedures for exclusion of data points, including whether blinded to treatment assignment.

There is also a need to develop guidelines for animal studies to handle missing values, which have the same potential to produce systematic bias as does attrition. In RCTs and observational studies of humans, multiple imputation is gaining favour.

## Conclusions and Recommendations: Building on the ARRIVE Guidelines

In biomedical science, clinical and animal studies must be of high quality to yield valid inferences regarding aetiology, pathophysiology, prevention, and treatment. Whole animal experiments and RCTs work hand-in-hand to achieve these goals. Animal studies have the ability to unravel biological mechanisms and to suggest potential intervention strategies, whilst RCTs establish the efficacy of interventions on clinical outcomes and can provide invaluable evidence to establish aetiology. It stands to reason that both should adhere to the same rigor in study design and analysis.

In comparison with RCTs, however, the design and reporting of animal studies has received relatively little attention from the scientific community and thus has lagged in quality. The 2010 ARRIVE guidelines are an important first step toward transparency in reporting of animal studies, thus providing an incentive for researchers to improve their methods. Conducting follow-up surveys of animal studies, similar to those undertaken following the introduction of the CONSORT statement, will be important to gauge the effectiveness of ARRIVE in improving the quality of conduct and reporting of animal studies.

In addition, some areas of the ARRIVE guidelines need improvement, which we suggest should mirror the evolution of RCT quality as reflected in CONSORT. We have made specific recommendations in the areas of reporting of inclusion/exclusion criteria, randomization, blinding, adverse/unexpected events, sample size, and missing data (summarized in Table 2). We also believe that a registry of animal experiments would reduce publication bias, as do sites such as www.clinicaltrials.gov for human RCTs. Such steps are integral to improving the usefulness of whole animal experiments.

**Table 2:** Suggested modifications to the ARRIVE guidelines.

Subsection	Suggested Additions
**Methods**	
Participants/experimental animals	(a) Provide clear details of eligibility criteria in relation to strain, weight range, age range, etc. in Methods section of manuscript; (b) provide description of any run-in testing of suitability of animals for the main experiment; (c) clearly define primary and secondary outcome measures.
Sample size	(a) Provide justification of sample size selection and whether this was determined *a priori* (based on prespecified primary outcome).
**Randomization**	
Randomization sequence generation	(a) Report details of method of generating randomization sequence, including details of stratification if used.
Allocation concealment mechanism	(a) Provide details of whether the persons generating the randomization schedule were blinded to treatment.
Blinding	(a) Provide details of whether persons carrying out randomization, data collection, and data analysis were unaware of treatment group allocation/study hypothesis.
Statistical methods	(a) Indicate any subgroup analysis undertaken and details of how animals came to be included in the subgroup; b. report methods of accounting for non-independence of subjects (e.g. litter mates); c. Indicate whether intent-to-treat analysis was used.
**Results**	
Attrition	a. Provide a flow-chart of animals from source population through first allocation to the study, assignment to treatment group, to completion of experiment for each outcome measure; (b) provide description of criteria used for exclusion of animals/data points from analysis and whether these were determined *a priori*; (c) provide explanations for discrepancies in numbers between experiments/outcome measures (attrition, missing data); (d) provide explanation of process for reviewing erroneous data and whether this was undertaken blinded to treatment group.
Baseline data	Provide details of the animals used, including species, strain, sex, developmental stage, and weight for each experimental group (preferably in tabular form).
Numbers analysed	(a) Report number of animals in each group included in each analysis and whether this was by original assigned groups.

## Abbreviations

ARRIVE: Animal Research: Reporting In Vivo Experiments
CONSORT: Consolidated Standards of Reporting Trials
RCT: randomized controlled trials
